# Long-range linkage effects in adapting sexual populations

**DOI:** 10.1038/s41598-023-39392-z

**Published:** 2023-08-01

**Authors:** Igor M. Rouzine

**Affiliations:** Sechenov Institute of Evolutionary Physiology and Biochemistry, Russian Academy of Sciences, Saint-Petersburg, Russia 194223

**Keywords:** Evolutionary genetics, Evolutionary theory, Population genetics, Computational biology and bioinformatics, Evolution

## Abstract

In sexual populations, closely-situated genes have linked evolutionary fates, while genes spaced far in genome are commonly thought to evolve independently due to recombination. In the case where evolution depends essentially on supply of new mutations, this assumption has been confirmed by mathematical modeling. Here I examine it in the case of pre-existing genetic variation, where mutation is not important. A haploid population with $N$ genomes, $L$ loci, a fixed selection coefficient, and a small initial frequency of beneficial alleles ${f}_{0}$ is simulated by a Monte-Carlo algorithm. When the number of loci, *L*, is larger than a critical value of ${\text{4log}}^{2} \left( {Nf_{0} } \right),$ simulation demonstrates a host of linkage effects that decrease neither with the distance between loci nor the number of recombination crossovers. Due to clonal interference, the beneficial alleles become extinct at a fraction of loci $1-2\mathrm{log}\left(N{f}_{0}\right)/{L}^{0.5}$. Due to a genetic background effect, the substitution rate varies broadly between loci, with the fastest value exceeding the one-locus limit by the factor of ${[{L}^{0.5}/\mathrm{log}\left(Ns\right)]}^{0.75}.$ Thus, the far-situated parts of a long genome in a sexual population do not evolve as independent blocks. A potential link between these findings and the emergence of new Variants of Concern of SARS-CoV-2 is discussed.

## Introduction

Humans are heterozygous at millions of genomic sites, loci. The average difference between an individual's genome and the consensus genome is estimated at 20 million base pairs, or 0.6% of the total of 3.2 billion base pairs. The invention of the new methods of full-genome DNA sequencing caused the emergence of the field of genomics and proteomics dedicated to the quantitative aspects of genetic diversity and gene expression at a large number of loci. To describe and visualize the genetic complexity, various computational methods have been developed including phylogenetics, the principle-components analysis, the cluster analysis. Among them, mathematical modeling of evolution stands out as a tool of a high predictive power. Modeling allows to connect, in the most direct and reproducible fashion, the assumptions about the dominant factors of evolution to the predictions for the observable parameters of genetic diversity and evolutionary dynamics.

The assumptions and simplifications of models vary broadly depending on the systems studied and the questions asked. Two distinct groups of models and methods have been applied to animal populations and microbial populations. The classical one-locus and two-locus models that neglect interaction with the other loci in genome dominate the way in which many evolutionary biologists think about the evolution of higher organisms. In contrast, monocellular eukaryotes, viruses, and bacteria that are characterized by an extremely high genetic diversity and ultrarapid evolution, are often described by asexual or partly sexual population models that include explicitly large numbers of interacting loci. Analysis of the evolutionary dynamics of multi-locus models is more complex than one-locus and two-locus models and relies either on Monte-Carlo simulation^1,2^ or the advanced mathematical methods of statistical physics^3–10^.

The heavy mathematical artillery is required, because the evolution of many different loci is coupled. Two kinds of interference effects exist. One kind, not considered in this article, is epistasis arising from biological interaction of different loci, including protein–protein interactions or the interactions of gene regulation network^8,11–14^. The second type of interference, which is the focus of the present article, is the effects originating from the common ancestry of different loci, including clonal intereference and background selection. The effect of competition between clones with beneficial mutations at different sites was first described by Fisher^15^ and Muller^16^. Later, Hill and Robertson provided another argument showing that the action of selection at different sites is not independent^17^. Both effects were shown to be equivalent by Felsenstein^18^ and will be referred to below as “clonal interference”^3^. Linkage effects slow down adaptation^4,7^, increase accumulation of deleterious alleles^19^, and change the statistical shape of genealogical tree^5,6,20^. The focus of the present work is on clonal interference and “background selection”, the last term referring to the fact that selection acts at the level of whole genomes, and not separate loci.

In sexually reproducing organisms and viruses with frequent recombination, linkage effects are partly compensated by recombination between parental genomes. A fundamental fact of genetics discovered by Morgan is that frequent recombination destroys allelic associations, so that alleles at far-spaced loci segregate independently. Conventional wisdom tells us that all the other linkage effects between far-situated loci must vanish as well. Models of long-term sexual evolution depending on new mutation events confirm this expectation^21–23^. Assuming that genome consists from independently-evolving blocks and applying the phylogenetic theory of asexual evolution to each block, these authors constructed a scaling argument expressing the length of each block, the lead of the traveling wave, and the average coalescent time in terms of the average adaptation rate. The analytic predictions have been confirmed numerically for two particular models of population in the presence of natural selection and mutation.

In the present work, I investigate linkage effects in a different biological scenario, when natural selection and recombination act on pre-existing beneficial alleles, and new mutations can be neglected. This model is appropriate, for example, when a population migrates to a new environment, or a virus was subjected to rapid mutation for a period of time. Then the fixation of pre-existing beneficial alleles does not depend on mutation de novo.

## Results

### Model

The evolutionary factors included in the model are directional natural selection, random genetic drift, linkage, and recombination. A sexually reproducing population is comprised of $N$ individual genomes (or $N/2$ diploid genomes without allelic dominance), where each genome has $L$ loci, and $L$ is assumed to be much larger than unit. In the beginning, each locus is assumed to have a fraction ${f}_{0}$ of beneficial alleles, with a fixed fitness benefit $s$. The initial state of a population is obtained by the generation of random and uniform distribution of alleles across all sites and individual genomes. The value of ${f}_{0}$ is assumed to be in interval $\frac{1}{Ns}\ll {f}_{0}\ll 1$.

The evolution is simulated in MATLAB™ using a Wright-Fisher process, in which the progeny genomes replace the parental genome. The logarithm of the average progeny number (log fitness) is given by the product of the number of beneficial alleles and the selection coefficient $s$ (Eq. 9, Section "Materials and Methods"). The random number of progeny for each genome is generated using a random number generator and the broken-stick algorithm. The total number of genomes $N$ does not change. With some probability $r,$ which is an input parameter of the model, a genome undergoes a number of random crossovers with another, randomly chosen genome. The average crossover number is $M.$ One of the two parents is replaced with the recombinant. Below I assume $r=1$, which corresponds to fully-sexual reproduction. Parameters $r$ and $M$ can be connected to the average number of crossovers between two sites ${r}_{2}$, which enters 2-locus models, as given by ${r}_{2} = rM/L$. Altogether, the model has 5 input parameters $(N, L, s, r, M)$ and the initial value ${f}_{0}.$

New mutation events are absent. Epistasis and allelic dominance are neglected, and a haploid population is considered. Indeed, as it is well-known in population genetics, a diploid population with $N/2$ genomes and without dominance is effectively haploid, with a double number of genomes $N.$ The details are given in section "Materials and Methods".

### Extinction of beneficial alleles depends on a single composite parameter

**Figure 1:**
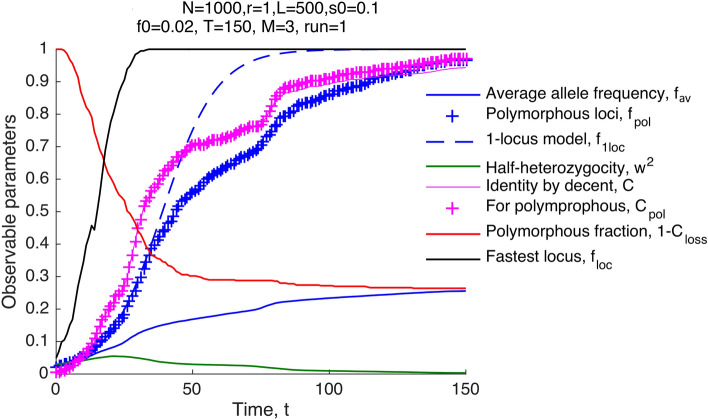
Dynamics of observables in the model with standing variation and the absence of mutation. Beneficial alleles become extinct at most loci. X-axis: Time in generations, $t.$ Y-axis: observable parameters calculated during simulation. The average frequency of beneficial alleles per locus per individual, ${f}_{av}$, the same value averaged over polymorphous loci only, ${f}_{pol},$ the prediction for ${f}_{av}$ of the deterministic one-locus model, ${f}_{1loc},$ half-heterozygocity ${w}^{2}=\langle f(1-f)\rangle ,$ the fraction of homologous pairs of loci with a common initial ancestor, $C,$ the same value for polymorphous loci, ${C}_{pol},$ the fraction of polymorphous loci, $1-{C}_{loss},$ and the largest of allelic frequencies among loci, ${\mathrm{max}(f}_{loc}).$ Parameter values are shown on the top. Parameters are defined in *Methods* and values are shown.

If the number of loci $L$ is sufficiently large, beneficial alleles at most loci become extinct. The fraction of remaining polymorphous loci, denoted ${1-C}_{loss}\left(t\right)$, decreases in time from 1 to at a low plateau (Fig. 1, red line).

This result differs strongly from the prediction of the single-locus model, in which multiple lineages per site are expected to reach fixation in the chosen parameter interval, and ${C}_{loss}$ is exponentially small. Indeed, in the single-locus model, the fixation probability of an allele is $s$, and the extinction probability is $1 - s$^24^. The probability of the extinction of $N{f}_{0}$ beneficial alleles present in the beginning is given by ${C}_{loss}\left(\infty \right)={(1-s)}^{N{f}_{0}} \approx {e}^{-N{f}_{0}s}$, which is exponentially small. Thus, in the parameter regime investigated, $N{f}_{0}s \gg 1,$ the one-locus model predicts that many alleles are fixed per each locus, and the loss of polymorphous loci due to genetic drift^25^ is negligible. Therefore, the observed loss is not a one-locus effect ocurring due to genetic drift, but is caused by competition between the clones of beneficial alleles expanding at different loci^3,15,16,18^.

**Figure 2:**
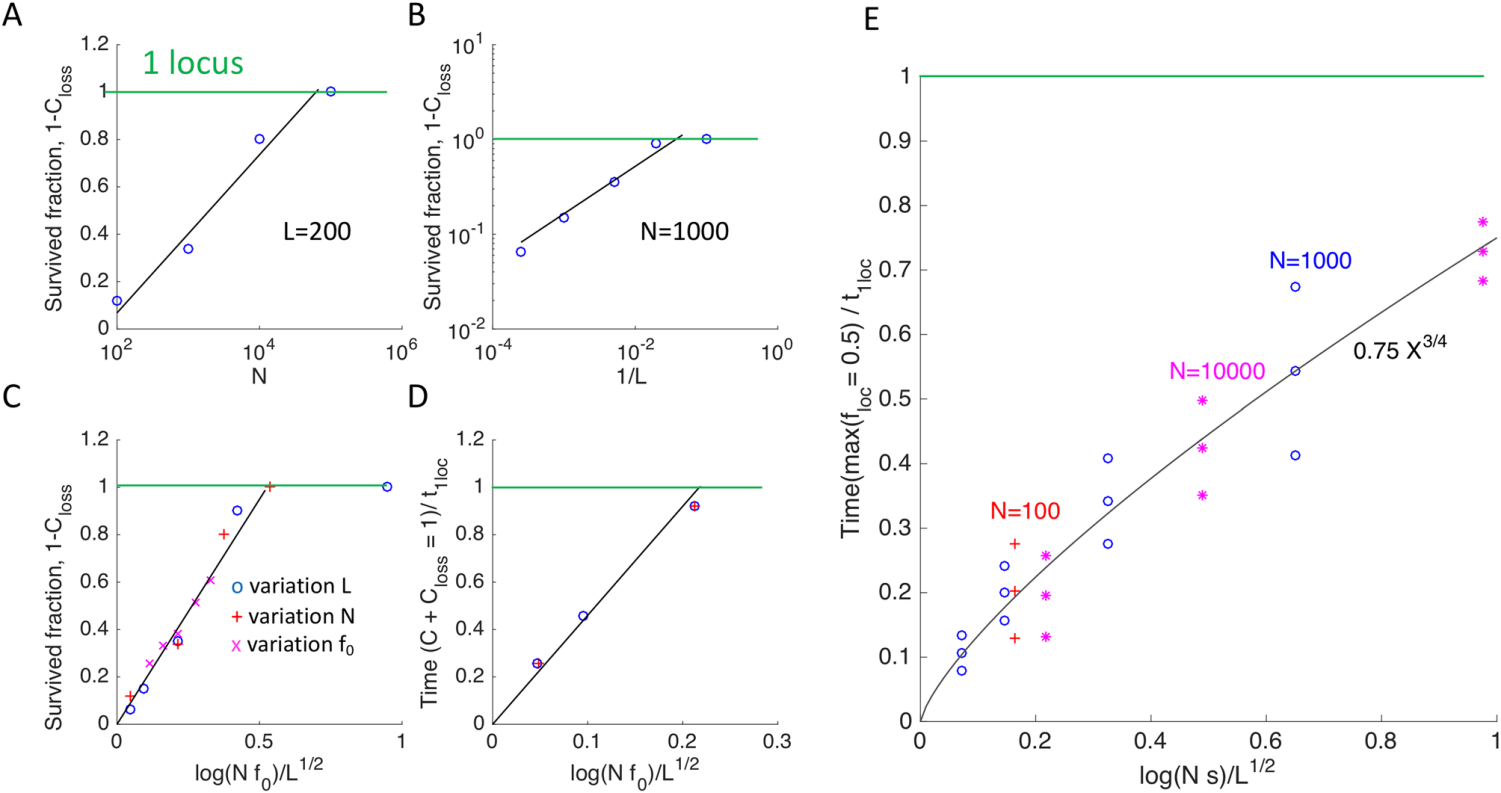
The observables depend mostly on a single composite parameter. (**A–C**). The locus fraction where beneficial alleles have survived and completed adaptation, $1-{C}_{loss}(\infty ),$ increases linearly with the natural logarithm of the population size, log $N,$ the inverse square root of the locus number, $1/\surd L,$ and a composite parameter, $\mathrm{log}(N{f}_{0})/\surd L$. Colored symbols , , and  correspond to the variation of model parameters $L, N,$ and ${f}_{0} ,$ respectively, where ${f}_{0}$>> 1/$Ns$. The green horizontal line shows the prediction of the one-locus model, ${C}_{loss}\approx 0$. (**D**) The time, $t,$ when the survived-loci fraction, $1-{C}_{loss}\left(t\right),$ equals the average identity by descent, $C\left(t\right),$ [intersection of red and pink curves in Fig. 1] scales linearly with $\mathrm{log}(N{f}_{0})/\surd L$ as well. **(E)** The time when the allelic frequency at the fastest locus reaches 50%, scales as a power ¾ of a similar parameter, $\mathrm{log}(Ns)/\surd L$. The symbol triplets show the mean and the 95% confidence interval. Colored symbols , , and  show different values of $N.$ The sensitivity to the variation of selection coefficient $s,$ crossover number $M$, and initial allele frequency ${f}_{0}$ is shown in Fig. 4 and Fig. S1. The default parameter values are $N=1000, L=200, {f}_{0}=0.02$ unless shown otherwise. The other parameters are as in Fig. 1.

Varying model parameters in simulation, I found out empirically that the fraction of loci with non-extinct alleles, $1-{C}_{loss}\left(\infty \right),$ depends mostly on a single composite parameter (Fig. 2A–C)

1 $$1 - C_{{loss}} = {\text{ }}\left[ {\begin{array}{*{20}l} {2.0\frac{{\log \left( {Nf_{0} } \right)}}{{\sqrt L }}} \hfill & {\quad 1 \ll \log \left( {Nf_{0} } \right){\text{ }}<0.5\sqrt L } \hfill \\ {1{\text{ }}} \hfill & {\quad \log \left( {Nf_{0} } \right){\text{ }}>0.5\sqrt L } \hfill \\ \end{array} } \right.{\text{ }}$$

Note the critical point, $\mathrm{log}(N{f}_{0})= 0.5\surd L$. If the population size is too large or the number of loci is too small, no significant loss of polymorphism is predicted. Intuitively, the clonal interference effects are expected to increase with the number of intefering loci, i.e., the length of genome $L$, just as the linkage effects on the adaptation rate increase with $L$^4^. The reason for a seemingly sharp transition remains to be investigated by analytic methods.

### The fastest adaptation rate among loci is much faster than in a single-locus model

Because most loci fail to complete adaptation, the average frequency of beneficial alleles per locus, ${f}_{av}\left(t\right)$, saturates far below 1 (Fig. 1, blue line). The dependence of average heterozygocity on time, ${2w}^{2}\left(t\right),$ is decreased accordingly (Fig. 1, green). The allele frequency averaged over remaining polymorphic sites, ${f}_{pol}\left(t\right),$ increases in the same general time range as the one-locus prediction. The time of half-fixation of polymorphous sites, ${t}_{50}$, is very close to the deterministic one-locus prediction, ${t}_{50}\approx {t}_{1loc}$ (Fig. 1)

2 $${t}_{1loc}=\frac{1}{s} \mathrm{log}\frac{1}{{f}_{0}}$$

In the range of parameters $s=0.025-0.2, L=200-2000, N=1000-\mathrm{10,000}$, the relative difference between ${t}_{50}$ and ${t}_{1loc}$ is between -0.11 and 0.14. Compared to the one-locus model prediction (blue dashed curve in Fig. 1), the dependence ${f}_{pol}\left(t\right),$ experiences a delay in the late phases of adaptation and has a noticeable random oscillation component (Fig. 1, blue ).

**Figure 3:**
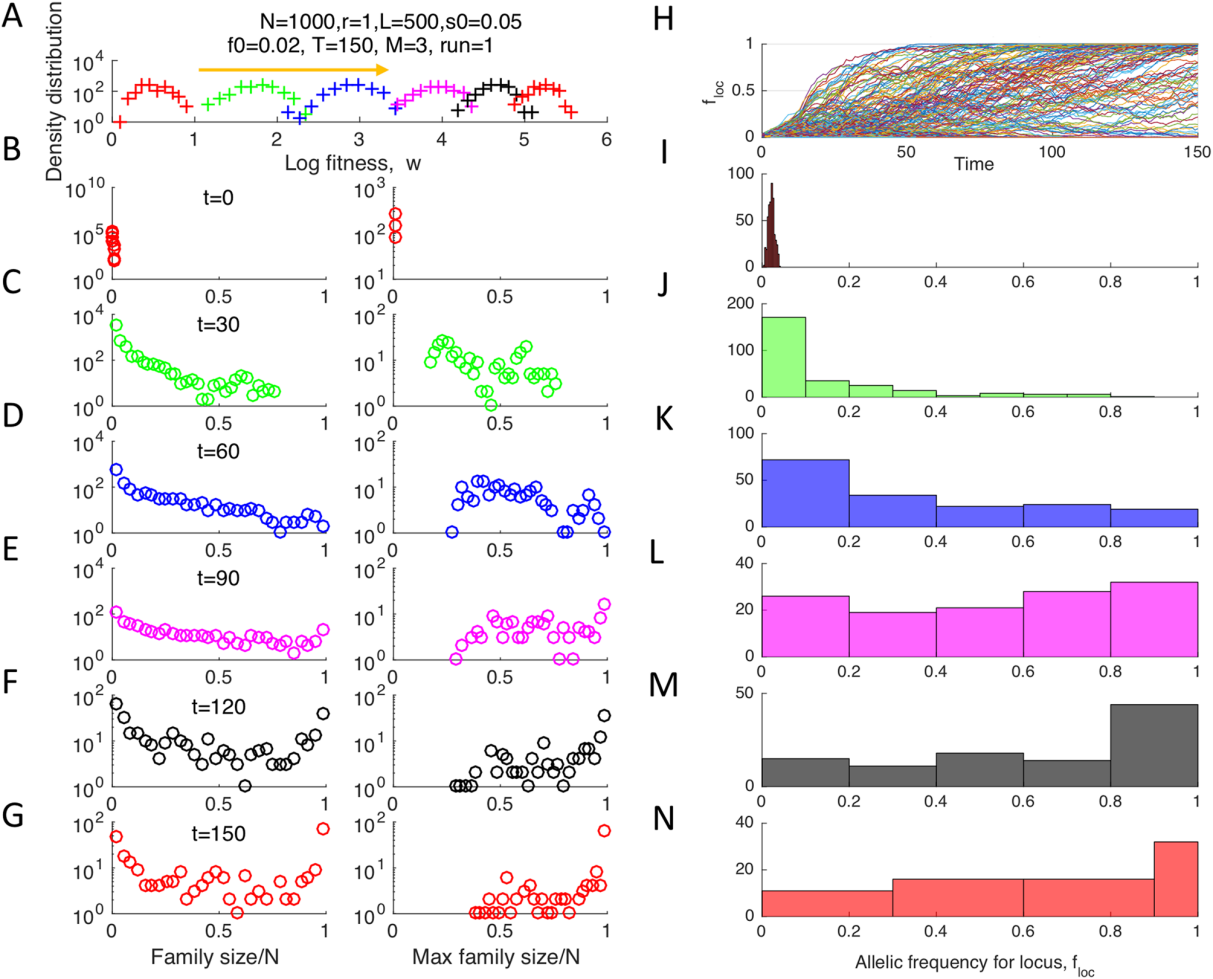
Traveling fitness wave and nonuniform dynamics of separate loci. (**A**) Distribution density of genomes in fitness at different time points shown in (B-G). (**B–G**) First column: Histograms of the family size defined as the number of sequences with the same initial ancestor at a locus. Second column: Only the largest family per locus is taken into account. (**H**) The average allelic frequency for each separate locus, ${f}_{loc},$ as a function of time. (**I–N**) Histograms of ${f}_{loc}$ across loci at different time points (shown). Parameters are as in Fig. 1.

At some loci, alleles accumulate much faster than predicted by the one-locus model (Fig. 1, black line). Indeed, the half-time of adaptation of the fastest locus, ${\mathrm{max }(t}_{loc})$, is much shorter than ${t}_{1loc}$ and increases as power ¾ of composite parameter $\frac{\mathrm{log}\left(Ns \right)}{\sqrt{L}}$(Fig. 2E). The ultrarapid evolution at some loci implies that the genome segments comprising these loci tend to have unusually high numbers of beneficial alleles. Indeed, the fitness of a genome is set to be proportional to that number. In other words, the broad variation in the evolution rate between loci with an identical selection coefficient demonstrates the existence of a strong background selection effect created by random recombination events. Recombination brings together different numbers of favorable alleles in different segments, and natural selection favors the fittest. The resulting distribution of genomes in fitness forms a traveling wave, well-known for both asexual and sexual populations^24^ (Fig. 3A). The new genomes at the high-fitness edge of the wave are born by recombination. They grow much faster in number than the average genome at the wave maximum, causing the wave to move forward^26^.

The population has a complex lineage structure that varies between loci. For a given locus, a lineage is determined as the set of individuals that have the same initial ancestor. The lineages all initially consists from a single individual, the founder (Fig. 3B), but their sizes grow in time, at different rates for different loci, and become distributed in a very broad range (Fig. 3C–G). The lineage size distribution between loci shifts in time towards larger lineages eventually occupying almost the entire population. If we take into account only the largest lineage for each locus, their size distribution looks similar but has a low cutoff increasing in time (Fig. 3B–G, column 2). The largest lineages grow to a half of the population at a much earlier time than ${t}_{1loc}$ in Eq. (2).

### Phylogenetic time scale depends only on the same composite parameter

Another quantity affected by linkage effects is the identity by descent, $C$, defined as the probability of a homologous locus pair to have the same initial ancestor. The average identity by descent averaged over all loci and over only polymorphous loci is almost the same (magenta line and magenta , Fig. 1). This result, again, differs from the single-locus prediction, where common ancestry is rare, $C\left(t\right)< {f}_{pol}^{2}\left(t\right)$, because each of the pair of loci must fall into the same growing lineage to have the same ancestor, and the size of each lineage relative to the population size is smaller than ${f}_{pol}\left(t\right)$. In contrast, in the present simulation, $C(t)$ is larger than ${f}_{pol}\left(t\right)$, which is larger than ${f}_{pol}^{2}\left(t\right)$.

At the time point ${t=T}_{2}$ such that $C({T}_{2})$ = $1-{C}_{loss}({T}_{2})$ , both quantities are close to a half, in a broad parameter range, as given by

$$C\left( {T_{2} } \right) \approx C_{loss} \left( {T_{2} } \right) \approx 0.5$$

The dependence of $T_{2}$ on model parameters can be interpolated by the formula

3 $$T_{2} \approx t_{1loc} \frac{{5.0 \log \left( {Nf_{0} } \right)}}{\sqrt L }$$

In other words, time ${T}_{2}$ is proportional to the same composite parameter that controls the fraction of successful loci, $1-{C}_{loss}\left(\infty \right),$ given by Eq. (1) (Fig. 2D). Time ${T}_{2}$ determined by Eq. (3) represents a proxy time scale of the phylogenetic tree. Although, at this time point, a population does not have a single ancestor for an average locus as yet, ${T}_{2}$ approximates the time to the most recent common ancestor by an order of magnitude.

### Observables depend weakly on the average number of recombination crossovers

**Figure 4:**
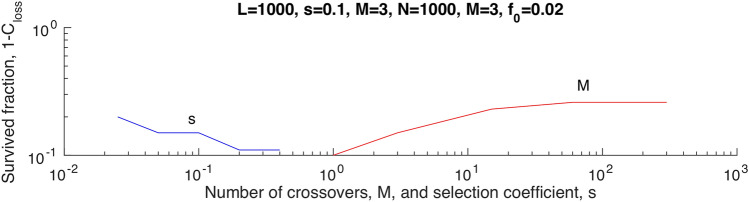
The fraction of loci that are not lost and complete adaptation are weakly senstive to the average crossover number. The default parameter values are shown on the top.

The above results in Figs. 1, 2, and 3 are weakly sensitive to the average crossover number, $M$. In its entire range of between $1$ and $L$, the fraction of loci that do not lose alleles, $1-{C}_{loss}\left(\infty \right),$ varies only by the factor of ~ 2 (Fig. 4). The variation of selection coefficient $s$ is rescaling units of time; otherwise, its effect is modest (Fig. 4).

### The absence of long-range linkage disequilibrium

**Figure 5:**
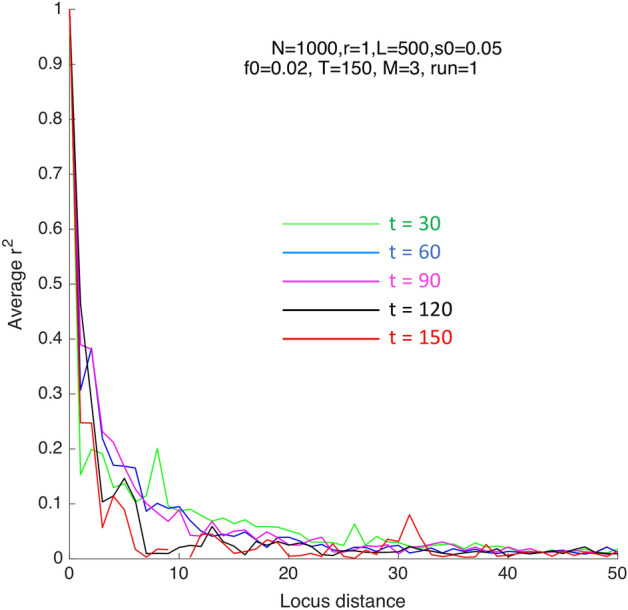
Linkage disequilibrium decays rapidly with the distance between loci in a genome. Y-axis: Pearson’s measure ${r}^{2}$, Eq. (4). The time points and parameters (shown) are the same as in Fig. 3. At $t=0$, linkage disequilibrium is identically zero due to the initially-random distribution of alleles.

A measure of linkage disequilibrium (LD) is Pearson correlator between allelic frequencies at two loci

4 $$r^{2} \left( {l_{12} } \right) = \frac{{\left\langle {\left( {f_{1} - \left\langle f \right\rangle } \right)\left( {f_{2} - \left\langle f \right\rangle } \right)} \right\rangle }}{{\left\langle {\left( {f_{1} - \left\langle f \right\rangle } \right)^{2} } \right\rangle }}$$

which is averaged over pairs of sufficiently heterozygous loci, $${2f}_{loc}\left(1-{f}_{loc}\right)> 0.1.$$ The value of $${r}^{2}$$ defined by Eq. (4) depends on time, as follows. Initially, $${r}^{2}\equiv 0$$ due to the random initial distribution of alleles set in simulation. After some time has elapsed, $${r}^{2}$$ becomes positive at any distance between loci (Fig. 5, $$t=30)$$. After passing through a maximum, $${r}^{2}$$ decreases in time again. At each next time point, $${r}^{2}$$ depends more and more sharply on the distance between loci, $${l}_{12}$$ (Fig. 5). In other words, alleles at far-situated loci segregate independently, as expected in the presence of recombination (Morgan’s law).

### Alleles are fixed inter-dependently

The fixation probability of an allele can be calculated as

5 $$P_{fix} = \frac{{{\text{log}}\left[ {1/C_{loss} \left( \infty \right)} \right]}}{{Nf_{0} }} \approx \frac{{1 - C_{loss} \left( \infty \right)}}{{Nf_{0} }}$$

In the parameter interval of interest, this value falls far below the 1-locus prediction, ${P}_{fix}^{1loc}=s$ (Fig. S1). Probability ${P}_{fix}$, Eq. (5), plateaus on the value of $s$ in the dilute limit of sufficiently small ${f}_{0}$ ^27^. Based on simulation, the transition point to the dilute limit ${f}_{0}^{dilute}$ decreases with $N$ and $L$. One can determine the transition point from condition ${P}_{fix}({f}_{0}^{dilute})=s$ and Eqs. (1) and (5), as follows

6 $$f_{0}^{dilute} \sim\frac{1}{Ns\sqrt L }\log \frac{1}{s\sqrt L },\, s\sqrt L \ll 1$$

The estimate from Eq. (6) agrees with the simulation results (Fig. S1).

### Phylogenetic tree varies between loci

In addition to calculating the phylogeny time scale ${T}_{2}$, we constructed the ancestral trajectory of a locus between individuals in real-time by recording the parentage of each individual locus and then tracing its ancestry back in time. Lineage of each locus jumps randomly between individuals due to recombination (Fig. S2A). If we straighten these trajectories and keep only the topology of coalescence and the coalescent times, we arrive at phylogenetic trees for different loci (Fig. S2B–D). As expected, the tree varies strongly between loci due to recombination, and the early branches are much shorter relative to late branches compared to Kingman’s coalescent constructed in the absence of selection^28^. The average density of coalescent events averaged over 10 runs and normalized to the prediction of the selectively-neutral model (*Methods*) decreases exponentially with time (Fig. S2E, F). This is because the coalescent event density is proportional to the inverse effective population size^28^, which is the size of the growing variant subpopulation. Importantly, the coalescent density is much larger than in the one-locus limit and increases with number of loci $L$. Thus, in agreement with the previous studies, uncompensated linkage in the presence of selection makes phylogenetic trees denser and changes their shape by making early stems shorter^5,6,20,29^ (Fig. S2E,F).

### Alleles “surf” between lineages

In addition to the ancestor number trajectory of a locus (Fig. S2A), one can also construct its fitness trajectory, by recording the fitness values of its ancestors (Fig. S2G). The fitness trajectory comprises alternating straight horizontal segments due to the clonal expansion connected to jumps caused by recombination. The jumps occur in both directions, but more often towards a genetic background with a higher fitness (Fig. S2G). This “allelic surfing” behavior was predicted for sexual populations analytically^27,30^ .

## Discussion

In the presence of standing variation, on moderate time scales, rapid evolution can occur in the absence of new mutations, and even much faster than due to mutation^26^. Such evolution based on standing variation and natural selection, with or without recombination, has been observed for poliovirus^31^ and VSV^32^. The present study considers a process of adaptation after minute quantities of beneficial alleles are generated in the beginning, for example, due to the change of external environment, or due to early mutation. If an allele is not lost to random genetic drift, its further accumulation is dominated by natural selection and recombination working together. The process continues, until all alleles are either fixed or lost.

Despite of the lack of observable LD for far-situated loci, simulation predicts the existence of strong long-range linkage effects encompassing the entire genome. The effects include the extinction of beneficial alleles at most loci, due not to random drift but to clonal interference, weak sensitivity of results to the number of crossovers, and ultrarapid evolution at some loci, even faster than in the independent-locus limit. The last observation implies the existence of genomic segments enriched in beneficial alleles over the average. Taken altogether, these results imply that far-situated genomic regions do not evolve independently, and recombination is not strong enough to break down linkage effects caused by selection acting, thus, at the level of a whole genome, as well as at the level of genomic segments.

If the locus number is decreased, or if the population size is increased, a transition to the independent-locus limit is predicted. The predicted dependence of all linkage effects on the population size $N$ is logarithmic (Fig. 2). For a genome of $200$ loci and ${f}_{0}=0.02, s=0.1$, the transition to the independent-locus regime can be observed already for 100,000 individuals. For a longer genome of $1000$ loci, they would evolve independently only for populations of ${10}^{12}$ individuals or larger, which is unrealistic for most species. A human or an animal population has millions of variable loci, of which a significant fraction is under natural selection, so that independent-locus models, probably, never work in most animals, except for rare mutations that are under very strong selection pressure.

The results of the present study carried out for the moderate-term evolution are in striking contrast to the previous findings for the long-term evolution driven by mutation, selection, and recombination, where genome was demonstrated to consist from quasi-independent blocks^21–23^. In my notation, the cited result for the average time to the most recent common ancestor has the form [^21^, Eq. 5]

7 $$T_{MRCA} \approx const\frac{M}{v}\log \left( \frac{Nv}{M} \right)$$

where $v$ is the average rate of long-term adaptation, defined as the fitness gain per unit time, *const* is a number on the order of 1, and the logarithm is supposed to be much larger than 1. In the present model, the proxy of ${T}_{MRCA}$ , by the order of magnitude, is ${T}_{2}$ in Eq. (3), and the adaptation rate is

8 $$v \approx const \frac{{sL\left( {1 - C_{loss} } \right)}}{{t_{50} }}$$

As already mentioned, the average time to a half-fixation for the loci that do not lose alleles, ${t}_{50},$ is always close to one-locus limit ${t}_{1loc}$. Substituting Eqs. (3) and (8) into Eq. (7), we get

$$\frac{M \mathrm{log}\left(Nv/M\right)}{s {\mathrm{log}}^{2}(N{f}_{0})}=const$$

The last equality is clearly false, because $M, N,$ ${f}_{0}$ and $s$ are independent parameters. Hence, Eq. (7) does not apply in the present case with pre-existing variation.

Note that the analytic argument in^21^ was developed and tested for the stationary long-term adapation. After the medium-term evolution ends with the loss or fixation of all initially-present alleles, further evolution requries a constant supply of new mutations. The derivation in^21^ is based on two statements: the assumption that a genome evolves as quasi-independent asexual blocks, and an expression for the time to the most recent common ancestor in terms of the average adaptation rate. The expression was based on the basic concept that the time to most recent common ancestor is the lead of the wave divided by the adaptation rate and was confirmed for various multi-locus models, both sexual and asexual. Therefore, it is likely that the quasi-independence assumption is the cause of the discrepancy.

**Figure 6:**
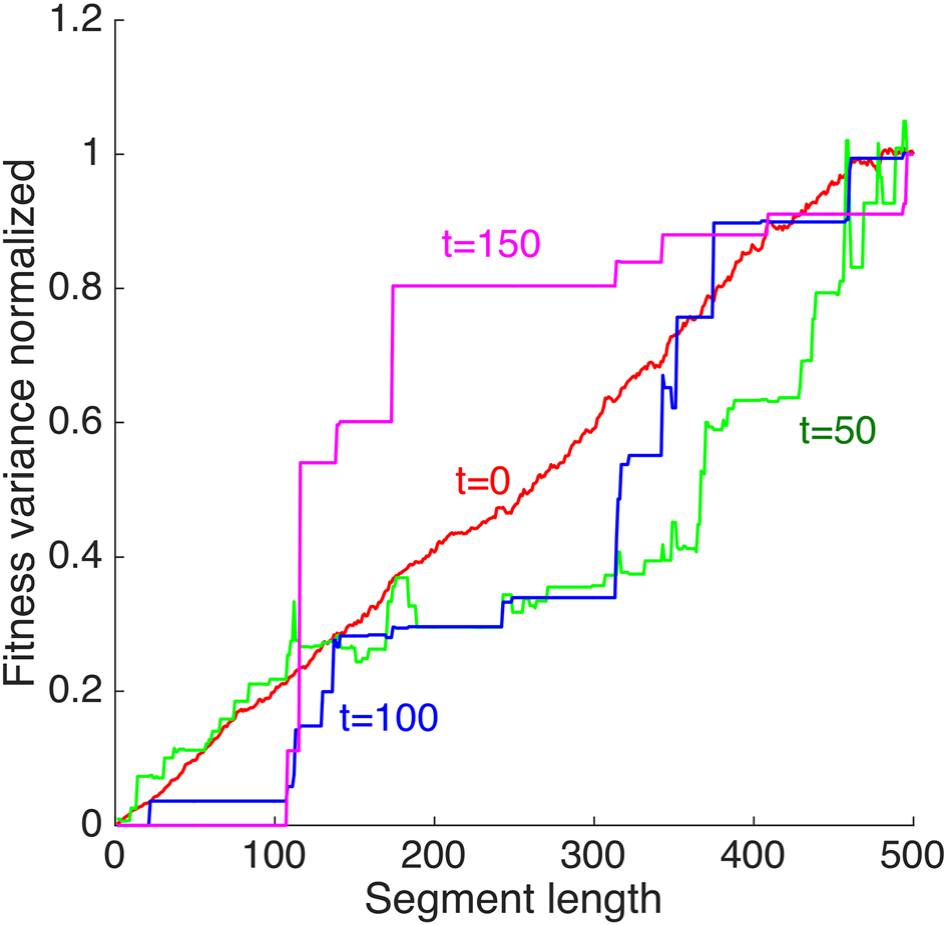
Non-linear dependence of genome segment variance on segment length. X-axis: the length of a genome segment starting from locus 1. Y-axis: Fitness variation between homologous genomic segments divided by the genome fitness variation at the same moment of time. A single Monte-Carlo run is shown. Parameters are, as in Fig. 1.

In other words, in the case of pre-existing variation and moderate-term evolution studied here, the genome does not evolve as a set of quasi-independent segments. That conclusion is indirectly confirmed by the results in Fig. 3 showing that beneficial alleles can form highly-fit genomes whose rapid growth outruns mixing of genomes due to recombination (Fig. 3). A recombinant that decreases fitness is not relevant for future generations. Furthermore, within one realization (Monte Carlo run), the fitness variance of a genomic segment normalized to the genome fitness is not linearly proportional to its length, but shows a complex step-like dependence (Fig. 6).

The results obtained in the present study might be potentially relevant for the viruses with frequent recombination, such as HIV, polio, or SARS-CoV-2. Similar to seasonal human coronaviruses or influenza virus, SARS-CoV-2 is constantly acquiring new mutations in its genome and has hundreds (if not thousands) of observably-diverse sites. Evolution is especially fast in receptor Spike protein, 5 replacements per year^33–36^. Two major reasons account for the high speed of evolution, as follows. Firstly, Spike has receptor-binding motives that affect transmission, and their evolution leads to the emergence of variants with enhanced transmissibility. Secondly, Spike contains epitopes, regions that are important for the immune response because of their involvement in binding of antibodies that can neutralize virus. Mutations in epitopes are a major factor that limits the virus recognition by the immune system and, hence, the durability of protection^37–39^.

A puzzle important for devising future vaccination strategies is the origin of the variants of concern (VOC) produced by large groups of new mutations that emerge all together at once^40–44^. Alternative theories of the emergence of VOCs^45^ include reverse zoonosis, the evolution within immunocompromised patients^46,47^ and the evolution in population pockets not covered by the genetic surveillance. Still another possibility is the fitness valley effect, a cascade emergence of compensating mutations following a primary mutation, an effect previously inferred for HIV and influenza^12,48^ and studied theoretically^49^.

Based on the present study, I may add yet another possible explanation. SARS-CoV-2, with its single-chromosome genome, has observable crossover recombination^50–52^. Hence, the large packages of mutations may emerge due to the combined effects of recombination and natural selection and represent the sequences comprising the fastest loci (Fig. 3H and J).

**Figure 7:**
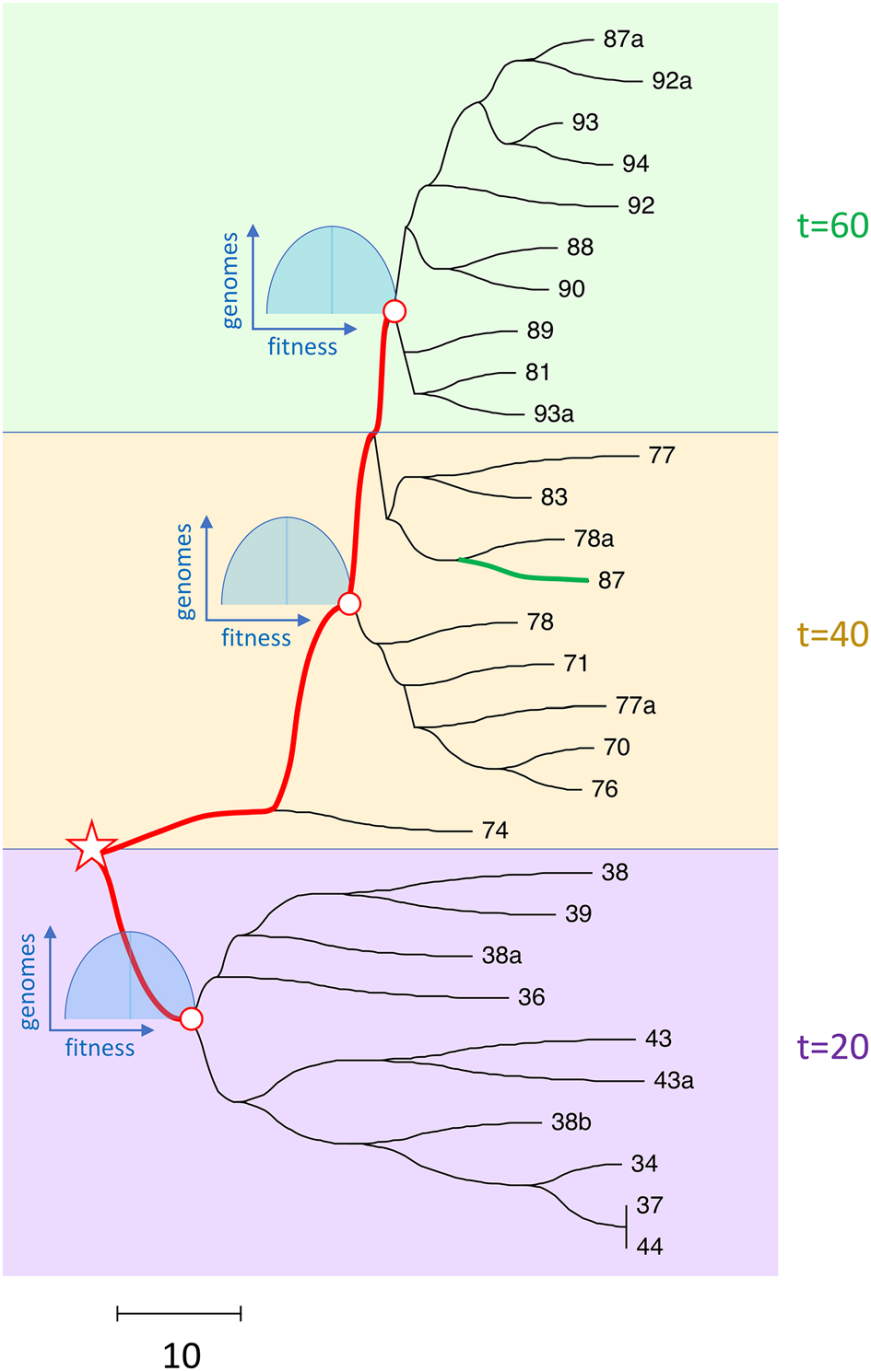
Phylogenetic tree at different time points has a polyphiletic structure with a common stem. Phylogeny of 10 simulated genomes sampled at three time points, $t=20, 40, 60$, is obtained by neighbor-joining analysis in MEGA11. Fat red line shows the stem connecting the subtrees. Open circles show roots of three subtrees emerging at the high-fitness edge of the genome distribution in fitness (shown). Number of beneficial alleles in a genome is shown in leaves. The scale of genetic distance measured in the number of differences is shown below. Model parameters are as in Fig. 1.

VOC are characterized by the sudden emergence of sister strains with large groups of mutations, occuring not on the background of the existing strain, but in parallel. This real-life observation can be compared to the tree predicted by the present simulation obtained by neighbor-joining analysis from a sample of 10 simulated sequences, at 3 time points each (Fig. 7). Note that genome samples obtained at different time points form separate subtrees (clades) growing on the same stem, the first two subtrees without apparent relation to each other. Each tree has an excess in the number of beneficial alleles compared to the previous tree. These features bear resemblance to VOC.

To understand the importance of recombination for SARS-CoV-2, we need to know the frequency of co-infected individuals among all the infected, which determines outcrossing probability $r$, an important input parameter entering the models of sexual populations^1,21,30^. For fully sexual reproduction considered in the present work, by the definition, $r=1.$ The outcrossing number for SARS-CoV-2 is presently unknown, but it could be larger than it seems due to the possibility of a co-infection during superspreading events^53,54^. Methods developed previously to quantify recombination from RNA sequence data for HIV could be re-applied to SARS-CoV-2^1,55^. The potential relevance of the present model for VOC of SARS-CoV-2, as well as a detailed analysis of SARS-CoV-2 in the light of this hypothesis, will be investigated elsewhere.

### Assumptions and limitations

(i) The model considered only variable sites under purifying selection. While a real population has many conserved sites and some selectively neutral sites as well, the existence of these sites has no effect on the evolutionary dynamics of the sites under purifying selection. Hence, these sites are not included into consideration explicitly.
(ii) We have investigated the case of a constant selection coefficient, but the results are expected to apply also for a sufficiently fast decaying distribution of selection coefficients, such as a Gaussian distribution. Distributions with long tails may have different properties, where the traveling wave is replaced by pairwise clonal interference^7^. The border case is the exponential distribution often observed in experiments on pathogens, which fact has been explained^56^. In this case, the present scenario with a fixed effective value of selection coefficient applies at sufficiently large population sizes^7^.
(iii) The model assumed homologous recombination for the two reasons, as follows. Firstly, recombination in viruses and organisms is similar in the sense that the vast majority of recombination events occur between homologous templates and do not include insertions or deletions. Secondly, when non-homologous recombination does occur, which effect is more frequent in viruses, the resulting progeny is often defective and, hence, not important for the evolutionary dynamics of a population.

## Conclusion

In sexual populations with pre-existing beneficial alleles, in an exponentially broad range of population size, recombination cannot suppress long-range linkage effects, including the excessive loss of beneficial alleles due to clonal interference, independence of observables on the average number of crossovers, and superfast evolution at some loci due to a genetic background effect. A potential link of these findings to the emergence of VOC of SARS-CoV-2 will be investigated elsewhere.

## Materials and methods

Consider a fully sexual population with $L$ loci comprised of $N$ individual genomes. Each locus has initially $N{f}_{0}$ alleles, ${1/Ns\ll f}_{0}\ll 1$, with fitness benefit $s\ll 1$. In each generation step, each genome undergoes random crossovers with another, randomly chosen genome, with average crossover number $M$, producing a recombinant genome. One of the two parents is replaced with the recombinant. Genome number $j{ }$ with $k_{j} { }$ favorable alleles is replaced with a random number of its copies distributed according to the polynomial distribution implemented by “broken stick” method, as follows. $N$ random points are generated uniformly within the interval $[0, N]$ broken into $N$ segments. The length of segment $j$ is proportional to the fitness of the corresponding genome ${w}_{j}$

9 $$w_{j} = \frac{{\exp \left( {sk_{j} } \right)}}{{\mathop \sum \nolimits_{j = 1}^{N} \exp \left( {sk_{j} } \right)}}$$

The number of random values that fall into segment $j$ are taken to be the number of his progeny in the next generation. Thus, the total number of genomes stays constant. New mutations are neglected, which is shown to be correct in the short-term in the presence of pre-existing genetic variation, both in simulation and experimentally^31,32^. Epistasis is absent; for epistatic analysis, see^12^ and references therein.

Input model parameters are the selection coefficient across loci, ${s=s}_{0}$, population size $N,$ outcrossing rate $r=1,$ number of loci $L,$ initial beneficial allele frequency ${f}_{0},$ total simulation time $t$, average number of recombination crossovers $M,$ and the seed number of the generator of pseudorandom numbers.

Parameter ranges studied are $s=\left[0.025, 0.4\right], L=[10, 4000]$, $N=\left[{10}^{2}, {10}^{5}\right], M=\left[1, 300\right], {f}_{0}=\left[0.0001, 0.02\right].$ The main focus is on the interval of ${f}_{0}$ such that $\frac{1}{Ns}\ll {f}_{0}\ll 1.$ The transition to dilute limit $N{f}_{0}s \ll 1$ when alleles are fixed independently is shown in Fig. S1.

## Supplementary Information

Supplementary Figure 1.

Supplementary Figure 2.

Supplementary Legends.

## Data Availability

The simulation code is available at https://github.com/irouzine/Strong-linkage-in-sex.
