# Supplementary figures and images for: Long-range linkage effects in adapting sexual populations
Source: Sci Rep. 2023 Aug 1;13:12492. doi: 10.1038/s41598-023-39392-z (PMC10393966; doi:10.1038/s41598-023-39392-z)

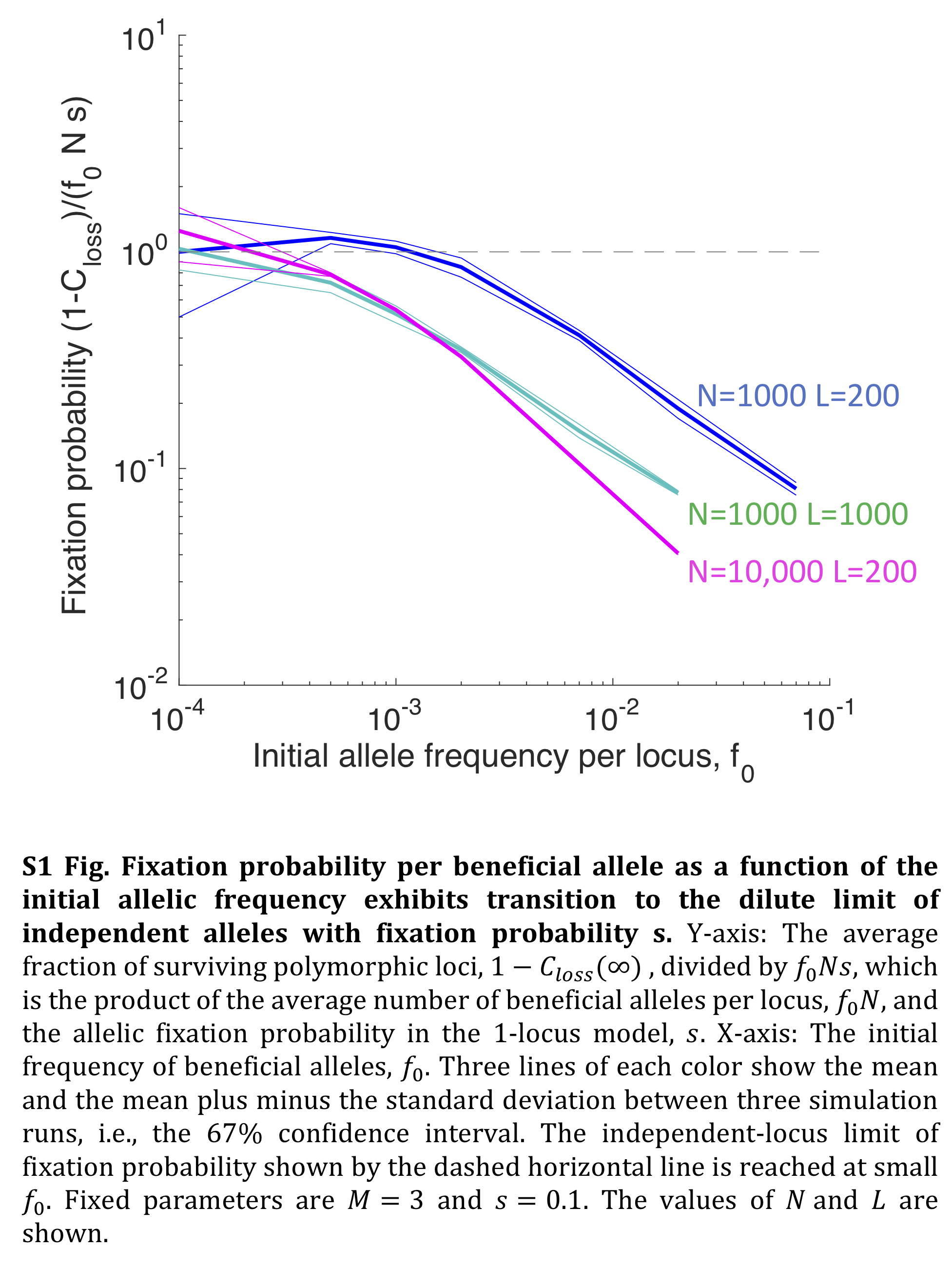

Supplement: Supplementary file 1 — Supplementary Figure 1. [file 41598_2023_39392_MOESM1_ESM.tiff]

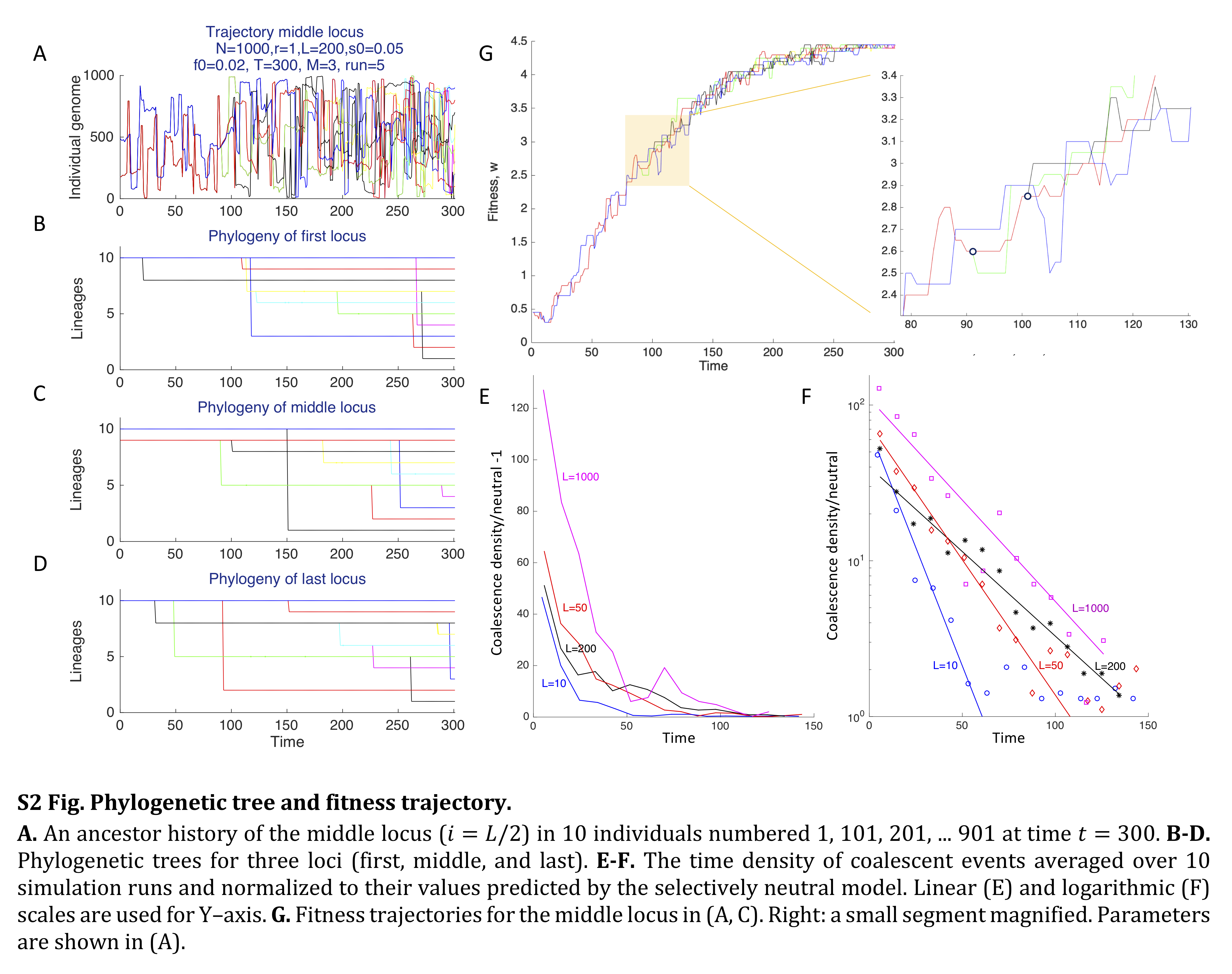

Supplement: Supplementary file 2 — Supplementary Figure 2. [file 41598_2023_39392_MOESM2_ESM.tiff]
