# Supplementary material for: Long-range linkage effects in adapting sexual populations
Source: Sci Rep. 2023 Aug 1;13:12492. doi: 10.1038/s41598-023-39392-z (PMC10393966; doi:10.1038/s41598-023-39392-z)
Supplement: Supplementary file 3 — Supplementary Legends. [file 41598_2023_39392_MOESM3_ESM.docx]

**Supporting Information**

**S1 Fig.** **Fixation probability per beneficial allele as a function of the initial allelic frequency exhibits transition to the dilute limit of independent alleles with fixation probability s.** Y-axis: The average fraction of surviving polymorphic loci, $1-C_{loss}(\infty)$ , divided by $f_{0}Ns$, which is the product of the average number of beneficial alleles per locus, $f_{0}N,$ and the allelic fixation probability in the 1-locus model, $s$. X-axis: The initial frequency of beneficial alleles, $f_{0}$. The dependence is shown at three combinations of values of $N$and $L$. Three lines of each color shxow the mean and the mean plus minus the standard deviation between three simulation runs, i.e., the 67% confidence interval. The independent-locus limit of fixation probability shown by the dashed horizontal line is reached at small $f_{0}.$ Fixed parameters are $M=3$ and $s=0.1$.

**S2 Fig. Phylogenetic tree and fitness trajectory.**

**A.** An ancestor history of the middle locus ($i=L/2$) in 10 individuals numbered 1, 101, 201, ... 901 at time $t=300$. **B-D.** Phylogenetic trees for three loci (first, middle, and last). **E-F.** The time density of coalescent events averaged over 10 simulation runs and normalized to their values predicted by the selectively neutral model. Linear (E) and logarithmic (F) scales are used for Y–axis. **G.** Fitness trajectories for the middle locus in (A, C). Right: a small segment magnified. Parameters are shown in (A).
